# Estimating Child Mortality Rate and its Trend in Hamadan Province, Western Iran from 1990 to 2016: Implications for Sustainable Development Goal

**Published:** 2018-03-07

**Authors:** Younes Mohammadi, Rashid Heidarimoghadam, Bistoon Hosseini, Mohammad Babamiri, Azita Nikravesh, Masoumeh Javaheri, Babak Moeini

**Affiliations:** ^1^Modeling of Noncommunicable Disease Research Center, Hamadan University of Medical Sciences, Hamadan, Iran; ^2^Department of Epidemiology, School of Public Health, Hamadan University of Medical Sciences, Hamadan, Iran; ^3^Research Center for Health Sciences, Hamadan University of Medical Sciences, Hamadan, Iran; ^4^Kermanshah Province Electricity Distribution Company, Kermanshah, Iran; ^5^Social Determinants of Health Research Center, Hamadan University of Medical Sciences, Hamadan, Iran; ^6^Deputy of Social Affairs, Hamadan University of Medical Sciences, Hamadan, Iran; ^7^Deputy of Health, Hamadan University of Medical Sciences, Hamadan, Iran

**Keywords:** Infant mortality/trend, Child mortality/trend, Conservation of natural resources, Iran

## Abstract

**Background:** Child mortality is one of the major health indices and the main targets in sustainable development goals. This study aimed to estimate child mortality rate and assess the progress toward sustainable development goals in Hamadan Province, western Iran and its districts from 1990 to 2016.

**Study design:** A cross-sectional study.

**Methods:** We used two data sources including death registration system (DRS) and summary birth history data (SBH) of 2010 census for estimating child mortality rate. SBH data was analyzed by Maternal Age Cohort and Maternal age period methods. To obtain the final trend with 95% uncertainty, we used Bayesian Penalized B-Spline.

**Results:** At provincial level, child mortality rate reduced by 82% from 1990 (97 per 1000 live births) to 2016 (16 per 1000 live births). At district level, in 2016, the highest child mortality rate belonged to Toyserkan and Kaboodarahang districts with 18 per 1000 live births, and the lowest child mortality rate belonged to Hamadan and Razan districts with 12 per 1000 live births. The highest and the lowest reduction rate from 1990 to 2016 belonged to Razan and Kaboodarahang districts, respectively.

**Conclusions:** The rate of child mortality has declined massively at both provincial and district levels. However, disparity existed among districts of Hamadan Province. The level of maternal education and income level was associated with disparity.

## Introduction

One of the preferred tools for examining population health status is to investigate child mortality rate. The researchers and policy-makers use this indicator for monitoring health and economic situation and examine the effectiveness of interventions in communities. Moreover, mortality data account for cornerstone of global burden of disease (GBD) and risk factor studies ^1,2^.

Despite mass interventions to reduce child mortality, the rate of child mortality is still high. In 2016, about six million children died before reaching age five year ^3^. Due to the importance of child mortality, the reduction of child mortality rate has always been one of the major targets for world health systems. The fourth goal of the millennium development goals (MDG) was to reduce under-five mortality rate (UFMR) by two-thirds during 1990 to 2015^4^. After MDG, the target for reducing UFMR continued in the case of sustainable development goals (SDG), in which its third goal aimed at the reduction of UFMR to less than 25 per 1000 live births by 2030^5^. Accordingly, national and international researchers including United Nations Inter-agency Group for Child Mortality Estimation (IGME) and Institute for Health Metrics and Evaluation (IHME) publish the yearly estimates of child mortality for all countries ^3,6^. The estimates are very useful for countries, but the estimates are published at national level only and not sub-national and district level, therefore, we cannot explore inequality within the countries and the provinces.

The studies confirm Iran's success, and even some provinces achieve MDG and SDG, but our information on status at district level is indistinct^7^. The main cause for our ignorance is the deficiency of death registration system (DRS). The studies endorse on under-registration and misclassification of DRS in Iran^8,9^. Hamadan Province is located in western Iran. In a study that estimated child mortality rate for Iranian provinces, Hamadan had achieved both MDG and SDG in 2015^7^. However, due to the above-mentioned problems of DRS, there is not a clear picture of the status of the provinces' districts according to MDG and SDG.

In this study, we aimed to estimate the trends and levels of UFMR and to quantify the magnitude of the reduction rate in Hamadan Province, western Iran with its districts, from 1990 to 2016, and use it as baseline analysis to measure progress toward MDG and SDG.

## Methods

### Study design

In this cross-sectional study, child mortality rate was the risk of death from birth to five years of age, stated per 1000 live births.

### Study area

Hamadan Province is one of the 31 provinces of Iran and the capital is the city of Hamadan. Regarding the 2016 census, the province had a population of approximately 1.740 million people. The province has nine districts including Hamadan, Toyserkan, Nahavand, Malayer, Asad Abad, Bahar, Famenin, Razan and Kabudarahang ^10^.

### Data sources

Three sources are available for child mortality. DRS is the recommended source by WHO. Most countries lack such system; therefore, they use two alternative sources. Complete Birth History (CBH) and Summary Birth history (SBH). The necessity of using these two sources is to perform a survey. In CBH, the detailed question on birth history such as date of birth, order of birth, sex of infant etc. are asked from the reproductive age women. Since the questions are long and require intense labor, CBH questions are incorporated into small surveys such as Demography and Health Survey (DHS).

Due to problems related to recall bias and long work, researchers suggest using SBH. In this method, no information item is asked from the reproductive age women. The summary birth history questions are called the indirect method which includes only two questions: how many children have ever been born to each mother? And how many of those children are alive now? The method applied mother's age as a proxy for children's age to estimate child mortality rate. In this method, no time information is required. Therefore, the problem of recalling bias related to CBH is not Problematic. The questions of SBH are incorporated into censuses and DHS.

However, in this study, we used two data sources including DRS and censuses 2005 and 2010 for estimating UFMR in Hamadan Province. However, the data for mortality in Hamadan was not available for all the years. DRS Data was available from 2009 to 2015. The second child mortality data source was censused which included SBH questions. These data were available for 1996, 2006 and 2011 censuses. We provided the detailed explanations of the data sources elsewhere ^11^.

### Statistical analysis

First, we analyzed SBH questions using two methods: maternal age cohort (MAC) and maternal age period (MAP). These methods are demographic methods which indirectly estimate child mortality rate for different years. In MAC method, based on seven age groups of mothers (15-19, 20-24….45-49), we have seven estimates for UFMR for seven time periods, while in the MAP method, because the method estimates child mortality rate for each year prior to survey, we have 25 estimates for 25 years before the survey. In the next stage, we smoothed two estimates using Loess regression, which is a locally weighted scatter plot smoothing method and does not require any assumption for fitting data ^12,13^. In this regard, a paper was published describing these methods in detail ^11^. Finally, we used Bayesian Penalized B-Spline to produce final trend with 95% uncertainty. By using the Rstan package in R software and the Hamiltonian Monte Carlo method, posterior distribution was produced and then the parameters with confidence intervals were calculated ^14^.

## Results

**Figure 1:**
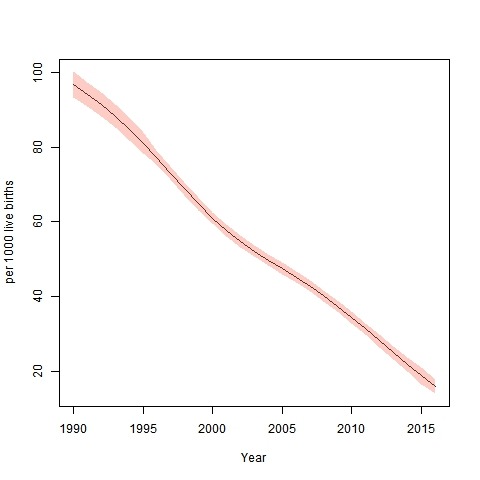
Child mortality rate in Hamadan province from 1990 to 2016

Figure 1 demonstrates the 26 yr trend of UFMR at provincial level. The rate is 97 (95%CI: 93-100) per 1000 live births in 1990, 61 (95%CI: 59-63) per 1000 live births, in 2000, 34 (95%CI: 33-36) per 1000 live births in 2010 and 16 (95%CI: 14-18) per 1000 live births in 2016. This trend indicates 82% reduction in child mortality rate at provincial level during 1990 to 2016. Accordingly, median Annual Reduction Rate (ARR) for the 27 years was over 5%.

Moreover, at district level, there is a substantial reduction in child mortality. However, the degrees of disparity between districts are present. In 2016, Hamadan (The capital) and Razan districts with 12 per 1000 live births had the lowest child mortality rate, and Toyserkan district with 18 per 1000 live births has the highest child mortality rate in the Province. The other districts were located between these two rates (Table 1).

**Table 1:** Child mortality rate with 95% uncertainty in districts of Hamadan Province, western Iran from 1990 to 2016

**District**	**1990**	**2000**	**2010**	**2016**
Asadabad	83 (75 to 91)	38 (35 to 40)	28 (25 to 30)	16 (13 to 19)
Bahar	90 (84 to 96)	69 (66 to 73)	43 (39 to 46)	17 (14 to 21)
Famenin	195 (184 to 205)	82 (77 to 86)	44 (40 to 49)	18 (13 to 23)
Hamadan	77 (74 to 80)	62 (59 to 64)	35 (33 to 37)	12 (9 to 15)
Kaboodarahang	78 (75 to 81)	65 (62 to 67)	35 (32 to 37)	18 (16 to 21)
Malayer	78 (75 to 82)	47 (45 to 49)	26 (23 to 28)	14 (12 to 17)
Nahavand	142 (136 to 148)	67 (63 to 70)	28 (24 to 31)	14 (11 to 17)
Razan	134 (128 to 139)	65 (61 to 68)	26 (23 to 29)	12 (9 to 15)
Toyserkan	142 (137 to 147)	98 (94 to 102)	48 (44 to 51)	18 (15 to 22)
Province	97 (93 to 100)	61 (59 to 63)	34 (33 to 36)	16 (14 to 18)

Total reduction from 1990 to 2016 showed that Razan with 90% and Kaboodarahang with 76% had the highest and the lowest ARR, respectively (Table 2). On the other hand, median ARR for Bahar with 4% was the lowest and for Nahavand and Razan with over 8% was the highest ARR (Table 2).

**Table 2:** Median annual reduction rate (ARR) and total reduction of Child mortality rate in districts of Hamadan Province, western Iran from 1990 to 2016

**District**	**ARR**	**Total reduction**
Asadabad	-0.05	-0.80
Bahar	-0.04	-0.80
Famenin	-0.06	-0.85
Hamedan	-0.03	-0.84
Kaboodarahang	-0.05	-0.76
Malayer	-0.06	-0.81
Nahavand	-0.08	-0.90
Razan	-0.08	-0.90
Toyserkan	-0.07	-0.87
Province	-0.05	-0.82

## Discussion

In this study, we estimated the levels and trends of child mortality for Hamadan Province and its districts for a long period of time from 1990 to 2016. According to the dysfunctionality of the death registration system in Iran including Hamadan, we decided to use two sources with available district data, namely DRS and census 2010. We did not use complete birth history questions because the questions were collected at provincial level, not at district level.

However, our results indicate the impressive reduction in UFMR in Hamadan Province. At provincial level, the 82% reduction (97 per 1000 live births in 1990 and 16 per 1000 live births in 2016) during 1990 to 2016 has occurred. Therefore, based on the third target of MDG, at provincial level, Hamadan has achieved two-thirds reduction in child mortality rate during 1990 to 2105. Furthermore, based on the second target of SDG, the countries needed to reduce child mortality rate to fewer than 25 per 1000 live births until 2030 to achieve that target, so that Hamadan Province could achieve SDG before this deadline. Another study confirm the results of the present study for Iran and some of its provinces regarding MDG and SDG ^7^. At national and sub-national levels, mortality rate of under-fives at provincial level was 19 (95% CI: 15 to 23) per 1000 live births in 2015, while the estimate at national level was 19 (95%CI; 18 to 20) per 1000 live births. The latest estimate disseminated by Global Burden of Disease (GBD 2016) 2016 Mortality Collaborators showed that child mortality rate at global level in 2016 was 38.42 (34.48 to 43.05) per 1000 live births, and this rate for Iran was 17.8 per 1000 live births (95%CI; 12.59 to 24.53) ^3^. These results confirm the success of Iran in attaining the third target of MDG in 2015, and its progress toward SDG before 2030.

Moreover, in all districts of Hamadan Province, a major reduction in child mortality rate occurred. The reduction rate from 1990 to 2016 for all districts was over 75%. However, Kaboodarahang with 76% reduction had the lowest percentage and Famenin with about 90% had the highest reduction rate. Therefore, all districts of Hamadan Province met MDG in 2015 and in terms of SDG; all districts of Hamadan Province have already achieved SDG. However, despite this pleasant news on child mortality rate status in Hamadan Province, there is a relatively wide disparity among districts. However, absolute difference between the highest and the lowest child mortality rate has been reduced over time. Absolute difference in 1990, 2000, 2010 and 2016 is 65, 60 and 22 per 1000 live births, respectively, while it reaches 6 per 1000 live births in 2016. The decreasing trends indicate the reduction of inequality among the districts over time. Health and socio-economic factors caused reduction and inequality in child mortality rate. Reduction in fertility rate increased mother literacy, improved access to healthy drinking water and deposit of wastewater, improvement of economic status were identified as non-health factors ^15-17^. On the other hand, increased coverage of health systems (such as health houses in rural areas and health centers in urban areas in Iran), increased coverage of vaccination, family physicians in rural areas, implementation of Integrated Management of Childhood Illness (IMCI) are stated as health factors affecting the reduction of child mortality ^18-22^. Based on the results of DHS in 2010, access to health services, literacy level, access to drinking water, vaccination coverage and other health and non-health factors were improved over time in Hamadan^23^. Moreover, a negative correlation exists between child mortality rate in the districts and years of schooling and wealth index of the districts. Generally, the districts with low literacy and wealth index had high child mortality rate.

Therefore, we recommend the policy-makers in Hamadan Province to take measures to reduce child mortality rate and inequality between provinces. These measures are the increased level of mother's education, increased economic status of people through job creation and increased access to health services through equal distribution of health and medical workers.

In this study, we encountered several limitations. First, we could not use DHS data, because the data was not gathered at district level, it was gathered at provincial level only. Moreover, for the recent years, we just used the death registration system data, which has a degree of under-registration, therefore, our estimates were somewhat affected by these values. Third, we did not examine the cause-specific mortality rate in this study which can help to identify major leading cause of death among children of Hamadan, therefore, examining the cause-specific mortality rate in future studies are suggested. In addition, because the estimates were affected by a number of databases, we suggest that researchers estimate and update child mortality rate in the next years using the newest datasets such as DHS 2015 and census 2016.

## Conclusions

During the 27 years, the mortality rate of children under five years of age in Hamadan Province was reduced impressively at both provincial and district levels, therefore, the districts met the world's goals such as MDG and SDG agendas already. However, the significant disparity among districts exists due to health and non-health factors. To reduce this disparity, health, social and economic measures should be taken in districts with high mortality rate.

## Acknowledgements

We would like to thank the staff of Health Deputy of Hamadan University of Medical Sciences, especially Miss. Ghazzanfarzadeh, for providing technical help to the study.

## Conflict of interest statement

The authors declare that there is no conflict of interest.

## Funding

The study (No. 9503181260) was financially supported by Vice-Chancellor for Research and Technology of Hamadan University of Medical Sciences.

## Highlights

- Child mortality rate in Hamadan Province has been reduced impressively.
- A relative wide gap exists among the districts of Hamadan Province.
- Health and socio-economic factors play roles in inequality among the districts.
